# Impact of synbiotics on gut microbiota during early life: a randomized, double-blind study

**DOI:** 10.1038/s41598-021-83009-2

**Published:** 2021-02-11

**Authors:** Nopaorn Phavichitr, Shugui Wang, Sirinuch Chomto, Ruangvith Tantibhaedhyangkul, Alexia Kakourou, Sukkrawan Intarakhao, Sungkom Jongpiputvanich, COLOR Study Group, Anundorn Wongteerasut, Kaouther Ben-Amor, Rocio Martin, Steven Ting, Orapa Suteerojntrakool, Chonikarn Visuthranukul, Punnapatch Piriyanon, Guus Roeselers, Jan Knol

**Affiliations:** 1 Department of Paediatrics, Phramongkutklao Hospital, Bangkok, Thailand; 2 Danone Nutricia Research, Utrecht, The Netherlands; 3 Danone Nutricia Research, Singapore, Singapore; 4 Nutritional Unit, Department of Pediatrics, King Chulalongkorn Memorial Hospital, Chulalongkorn University, Bangkok, Thailand; 5 Department of Pediatrics, Thammasat Hospital, Faculty of Medicine, Thammasat University, Bangkok, Thailand; 6 Laboratory of Microbiology, Wageningen University, Wageningen, The Netherlands

**Keywords:** Microbiome, Microbiota, Nutrition, Paediatric research, Applied microbiology, Microbial ecology, Colon

## Abstract

Human milk is considered the optimal nutrition for infants and found to contain significant numbers of viable bacteria. The aim of the study was to assess the effects of a specific synbiotic combination at doses closer to the bacterial cells present in human milk, on intestinal bifidobacteria proportions (relative abundance), reduction of potential pathogens and gut physiological conditions. A clinical study was conducted in 290 healthy infants aged from 6 to 19 weeks. Infants received either a control infant formula or one of the two investigational infant formulas (control formula with 0.8 g/100 ml scGOS/lcFOS and *Bifidobacterium breve* M-16V at either 1 × 10^4^ cfu/ml or 1 × 10^6^ cfu/ml). Exclusively breastfed infants were included as a reference. Analyses were performed on intention-to-treat groups and all-subjects-treated groups. After 6 weeks of intervention, the synbiotics at two different doses significantly increased the bifidobacteria proportions in healthy infants. The synbiotic supplementation also decreased the prevalence (infants with detectable levels) and the abundance of *C. difficile*. Closer to the levels in the breastfed reference group, fecal pH was significantly lower while l-lactate concentrations and acetate proportions were significantly higher in the synbiotic groups. All formulas were well tolerated and all groups showed a comparable safety profile based on the number and severity of adverse events and growth. In healthy infants, supplementation of infant-type bifidobacterial strain *B. breve* M-16V, at a dose close to bacterial numbers found in human milk, with scGOS/lcFOS (9:1) created a gut environment closer to the breastfed reference group. This specific synbiotic mixture may also support gut microbiota resilience during early life.

**Clinical Trial Registration** This clinical study named Color Synbiotics Study, was registered in ClinicalTrials.gov on 18 March 2013. Registration number is NCT01813175. https://clinicaltrials.gov/ct2/show/NCT01813175.

## Introduction

In early life, the infant’s gut microbiota of healthy breastfed infants is normally dominated by infant-type bifidobacteria such as *Bifidobacterium breve*, *Bifidobacterium bifidum* and *Bifidobacterium longum* subsp. *infantis*. These members of the gut microbiota make the infant gut more resistant to pathogen colonization^1^, improve certain vaccination responses^2^, support immune maturation and support gut barrier development^3^. However, not all infants’ gut microbiotas are dominated by *Bifidobacterium* species and some are even devoid of them^4,5^. Environmental factors such as mode of delivery, antibiotics and feeding patterns influence bifidobacterial colonization of the infant’s gut. Given the major role of infant-type bifidobacteria in structuring the gut microbiome in early life, it is important to support the colonization by relevant *Bifidobacterium* species^6^.

Opportunistic pathogens such as *Clostridium difficile, Clostridium perfringens,* enteropathogenic *Escherichia coli* (EPEC) and enteroaggregative *Escherichia coli* (EAEC) are often found in infants’ guts. *C. difficile* colonizes 10–70% of infants below 1 year of age^7^. *C. difficile* infections during infancy may not only cause diarrhoea but are also associated with higher risk of allergic diseases during early life^8^. Breastfeeding, known to reduce the prevalence of *C. difficile* in infants compared to formula feeding (14% vs 30%, respectively)^9^, also helps in prevention of infections and allergic diseases during early life^10,9^.

Human milk is considered the optimal nutrition for infants and contains a significant number of viable bacteria, which are an important source for vertical microbial transmission from mother to infant^11–13^. If this colonization route is disrupted, early life microbiota development may be impaired.

Human milk is estimated to contain about 10^3^–10^5^ bacterial cells/ml based on flow cytometry and quantitative polymerase chain reaction (q-PCR) methods^12,14–18^. The human milk microbiota is a taxonomically diverse community, to which, bifidobacteria contribute up to 10^4^ cells/ml^17,19^. *B. breve* is the most commonly isolated infant-type *Bifidobacterium* species from human milk^20^. It is one of the dominant members of the infant’s gut microbiota, involved in the metabolism of human milk oligosaccharides (HMOs) and the production of vitamins^21,22^. Non-infant-type bifidobacteria such as *B. animalis* subsp. *lactis,* isolated from diverse mammalian hosts, and *B. adolescentis*, normally found in the adult human gut, are genetically less equipped to metabolize HMOs^22^.

Synbiotics, a combination of probiotics and prebiotics that confers health benefits to the host^23,24^, offer an efficient way to mimic milk driven colonisation and formation of a *Bifidobacterium* dominated ecosystem in the infant gut^25^. Synbiotics containing short-chain galacto-oligosaccharides and long-chain fructo-oligosaccharides (scGOS/lcFOS) with a 9:1 ratio and *B. breve* M-16V, has been shown to restore the delayed bifidobacteria colonization in caesarean section (C-section) born infants^25^ and to improve the symptoms of IgE-associated atopic dermatitis^26^.

In addition, there is long and comprehensive tolerance and safety track record for the use of *B. breve* M-16V as a probiotic for infants, including infants with a very low birth weight.

The effects of probiotics or synbiotics are dose and strain dependent. The doses of probiotics and synbiotic used in previous studies in infants and children range from 10^8^ to 10^11^ cfu/day^27^.

As human milk contains relatively low numbers of viable bacteria (ranging from 10^3^ to 10^5^ cfu cfu/ml, about 10^6^–10^8^ cfu/day)^12^, it is important to understand the effects of different doses of synbiotics on the infant’s gut microbiota.

The primary objective of this study was to evaluate the bifidogenic effect of an infant formula containing synbiotics with two doses of *B. breve* M-16V (either 1 × 10^4^ cfu/ml or 1 × 10^6^ cfu/ml), in combination with scGOS/lcFOS (9:1) in healthy infants aged 6–19 weeks. The study also explored the effects of this specific synbiotics on pathogen reduction and gut physiological conditions in early life.

## Patients and methods

This was an exploratory, randomized, double-blind, controlled study conducted between May 2013 and September 2015 in Thailand. The protocol and all accompanying material provided to the subjects, such as information sheets or description of the study used to obtain informed consent, were submitted to the following ethics review committees: Institutional Review Board, Chulalongkorn University; Institutional Ethics Review Committee, Royal Thai Army Medical Department; Human Research Ethics Committee, Thammasat University. Approval from the three Ethics Committees was obtained before start of the study, and was documented in a letter to the investigators specifying the date on which the committee met and granted the approval.

Written informed consent was obtained from all parents/caregivers before inclusion in the study. The study was registered in ClinicalTrials.gov (March 18, 2013; #NCT01813175). The study was conducted according to ICH-GCP principles, and in compliance with the principles of the ‘Declaration of Helsinki’ (59th WMA General Assembly, Seoul, October 2008) and with the Thai laws and regulations. Inclusion criteria were a gestational age between 37 and 42 weeks, infant age 43–65 days, and exclusive formula feeding for at least 1 week (except for the breastfed reference group). Exclusion criteria were, malnutrition, weaned before inclusion, malformations, use of systemic antibiotics or anti-mycotic drugs within 4 weeks prior to study entry, gastroenteritis or diarrhoea in the last 2 weeks prior to study entry. Sample size calculation methods and randomisation and unblinding procedures are reported in detail in the “Supplemental Information and Methods” section.

**Figure 1:**
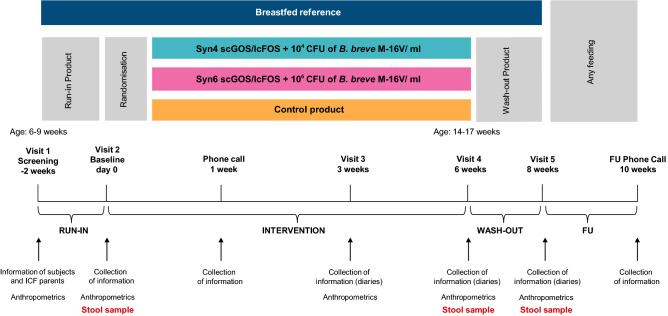
Study design.

Eligible infants in the formula-fed group started a 2-week run-in period with regular non-hydrolysed cow’s milk based infant formula (Nutricia, The Netherlands). Infants, who had successfully completed the run-in period, were randomized to receive the control formula or either one of the two investigational formula; control formula supplemented with 0.8 g/100 ml scGOS/lcFOS and *B. breve* M-16V at a dose of either 1 × 10^4^ cfu/ml (Syn4) or 1 × 10^6^ cfu/ml (Syn6) for 6 weeks. After the intervention period, infants received control formula for a wash-out period of 2 weeks. Non-randomized, exclusively breastfed infants were included as a reference (Fig. 1).

Stool samples were collected at baseline (after run-in period and before start of the intervention), Week 6 (after intervention) and Week 8 (after wash-out). Stool samples were collected by the parents into stool containers provided by the investigators. Samples were frozen at temperature of − 15 to − 20 °C immediately after collection by the parents and kept at this temperature until transport to the hospital and storage at − 80 °C. Fluorescent in situ hybridization (FISH)^28^ was used to assess the relative abundance (or proportion) of seven major gut bacterial taxanomic groups (Total *Bifidobacterium* species, *Bacteroides distasonis/Bacteroides fragilis*, *Eubacterium rectale/Clostridium coccoides, Lactobacillus*/*Enterococcus*, Enterobacteriaceae, *Atopobium*, *Clostridium histolyticum/Clostridium lituseburense*. The proportion or ‘relative abundance’ of these targeted taxonomic groups was measured by comparison with the total abundance of bacteria. In short, fixated fecal samples were hybridized with the taxon specific probes and then analysed using an automated Olympus AX70 epifluorescence microscope equipped with image analysis software. The relative abundance (or proportion) of cells belonging to a specific bacterial taxon was determined at 25 randomly chosen positions on each well by counting all bacterial cells using a DAPI filter set and by counting the targeted bacterial taxon using a Cy3 filter set.

Targeted microbiota quantification by q-PCR^29^ analyses was used to assess the abundance of *Bifidobacterium breve* and *Bifidobacterium breve* M-16V and the potential pathogens *Campylobacter jejuni*, *Clostridium difficile*, *Clostridium perfringens*, *Staphylococcus aureus*, Enteroaggregative *Escherichia coli* (EAEC), Enteropathogenic *Escherichia coli* (EPEC).

Short chain fatty acid (SCFA) and lactate were measured by Gas Chromatography (GC). Safety parameters (anthropometry, gastrointestinal tolerance, serious and non-serious adverse events) were also investigated. A detailed study scheme is illustrated in Fig. 1. A detailed description of the methods, including the oligonucleotide sequences of the primers and probes used for FISH and q-PCR analyses, is available in the “Supplemental Information and Methods”.

Danone Nutricia Research will grant data access, to researchers that meet the criteria for access to confidential clinical study data and are compliant with the DNR Clinical Trial Dataset Sharing policy.

### Statistical analysis

Analyses of continuous (and binary transformed) data were performed on the intention-to-treat (ITT) group. For safety data, the all-subjects-treated (AST) group was used. Continuous outcomes were modelled using a linear mixed-effect model for repeated measures (MMRM) including post-baseline and baseline measurements in the response vector, intervention, time and study site as fixed factors, intervention by time as interaction term and subject as a random effect. An unstructured covariance structure was used to model the correlation among repeated measurements. Supplemental Table S1 shows LS (Least Squares) estimates of differences in change from baseline between groups, Standard Error Estimates, 95% CI, and P-values for the linear mixed model key parameters measured at week 6 (*Bifidobacterium, Eubacterium*, pH, l-lactate, acetate, propionate, butyrate). Covariate assessment was performed for the analysis of *Bifidobacterium* in order to identify environmental factors (e.g. stool frequency, use of antibiotics and mode of delivery) that could potentially influence the estimate of treatment effect. The assessment was carried out by adding a single covariate into the linear mixed-effect model and evaluating the change in treatment effect estimate (10% or more change was considered relevant). In case the total number of non-detected measurements (or measurements below the limit of detection) for a specific parameter exceeded 30% of the data in at least one of the groups (for each comparison), the data were transformed into binary presence/absence (detected/non-detected) type of data. Prevalence of detected measurements was modelled instead, using a generalized linear mixed model (GLMM) with binomial distribution and a logit link function with study site as a fixed factor, intervention by time as interaction term, and subject as a random effect. Treatment comparisons were evaluated against control using a two-sided 95% confidence interval with corresponding p-value.

In addition to the univariate analyses performed for each (continuous or binary) parameter, Redundancy Analysis (RDA) constrained ordination was applied on the set of Hellinger-transformed fish data with the fish parameters as response variables and treatment as explanatory variable in order to assess the effect of treatment on the microbial assemblage composition. An ANOVA like permutation test^30^ was used to evaluate statistical significance of the treatment differences based on the resulting model. All analyses were performed using SAS (Enterprise Guide Version 4.3, SAS Institute, NC) except for RDA, which was performed using the ‘Vegan’ package in R (R software version 3.4.1, R Foundation for Statistical Computing, Vienna, Austria).

## Results

### Study population

**Figure 2:**
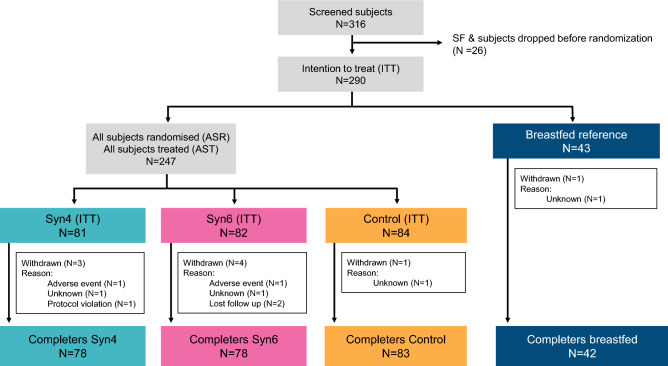
Study population composition. Screen Failure (SF) is defined as “potential subject did not meet one or more inclusion criteria. Intention to treat (ITT) includes every subject randomized to treatment assignment.

A total of 290 subjects were recruited, of whom 247 subjects were randomized into three intervention groups of which 239 subjects completed the study. The other 43 subjects were included in the non-randomized breastfed reference group of which 42 subjects completed the study (Fig. 2).

Demographic characteristics of the subjects recruited at the beginning of the study (Visit 1) are shown in Supplemental Table S2. The summary of study discontinuations (Supplemental Table S3) shows that most early terminations occurred at the end of the intervention period, just before or during the washout period (within week 8 and week 10).

This indirectly suggests that the study products were well tolerated by the subjects during the intervention. All infants were exclusively formula fed in the intervention groups and exclusively breastfed in the reference group. No statistically significant differences in gestational age, mode of delivery, gender, ethnicity and amount of milk intake were observed among the three intervention arms, resulting in three homogeneous groups.

### Effect of synbiotics on gut microbiota composition

No relevant differences were observed during the covariates assessment between the intervention effect estimate from the model including covariates from the list of predefined environmental factors and the intervention effect estimate from the model excluding the covariate (change in treatment effect estimate less than 10%).

**Figure 3:**
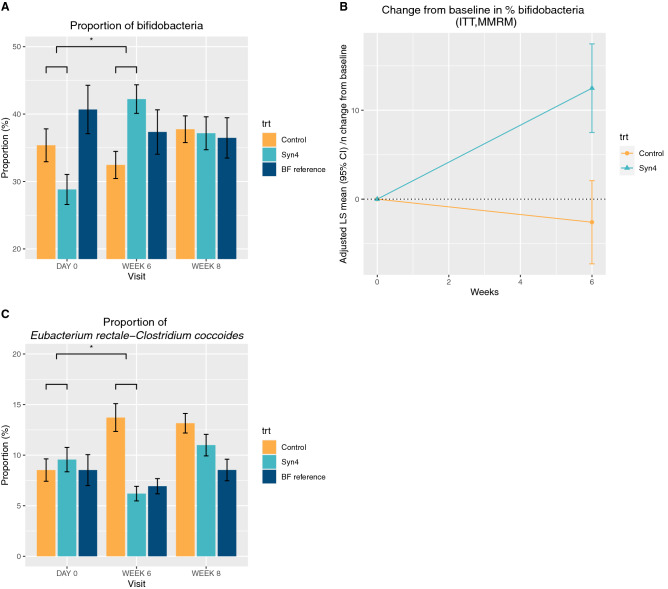
FISH analyses of fecal samples from the control treatment, Syn4 dose treatment and breastfed (BF) reference group. After 6 weeks the Syn4 dose treatment (trt) resulted in a significantly larger increase in the relative abundance (proportion) of bifidobacterial from baseline compared to control (**A**). (**B**) Shows adjusted LS mean (95% CL)/n change from baseline in relative abundance of bifidobacterial for the control and the Syn4 groups. Syn4 treatment lead to a significantly larger decrease in the relative abundance of *Eubacterium rectale–Clostridium coccoides* from baseline compared to control (**C**). A longitudinal linear mixed model was used with intervention, time, study site as fixed factors, intervention by time as interaction term and subject as a random effect. Data are expressed as mean ± SE. *Statistically significant difference in change from baseline between treatment groups (p-value < 0.05) as assessed by the linear mixed-effect model.

After 6 weeks of intervention, changes from baseline in the proportion of bifidobacteria (as measured by FISH) were significantly larger in both the Syn4 group (Fig. 3A,B) and the Syn6 group as compared to the control group (Supplementary Fig. S1A).

**Figure 4:**
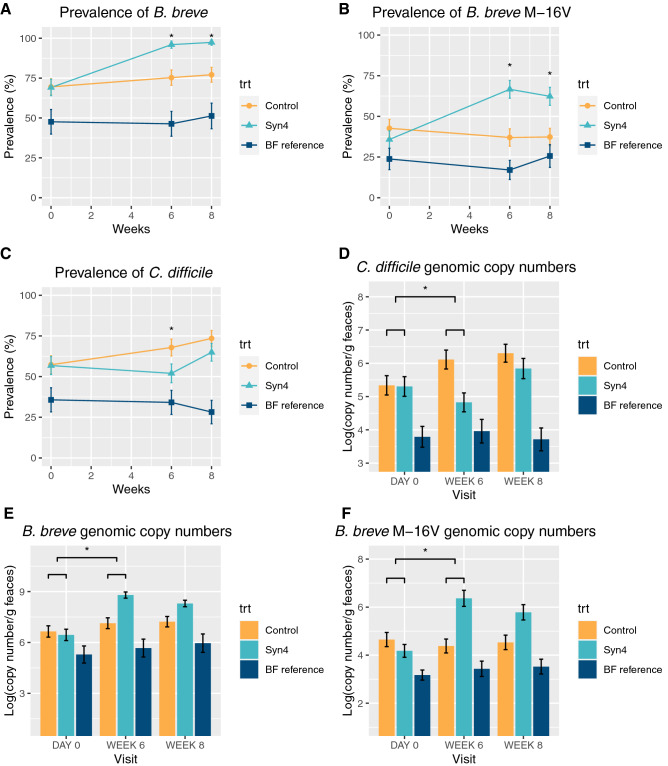
q-PCR analyses showed that the Syn4 dose resulted in a significantly larger increase in prevalence of (mean percentage of infants with detectable) *B. breve* (**A**) and *B. breve* M-16V (**B**) and a significantly larger decrease of *C. difficile* prevalence (**C**) as compared to control treatment. Detected *C. difficile* genomic copy numbers decreased significantly more in the Syn4 group compared to the control group (**D**). The increase in total amount of bifidobacterial copy numbers from baseline was only significantly larger (p = 0.0079) at Week 6 in the Syn4 group compared to control (**E**). Increase in the *B. breve* M-16V copy numbers from baseline was significantly larger in the Syn4 group compared to control at both Week 6 and Week 8 (respectively p < 0.0001 and p < 0.0001) (**F**). A generalized linear mixed model (GLMM) was used with intervention, time, study site as fixed factors, intervention by time as interaction term and subject as a random effect for the analysis of the binary transformed (detected/non-detected) data and the estimation of prevalence of detected values. A longitudinal linear mixed-effect model was used with intervention, time, study site as fixed factors, intervention by time as interaction term and subject as a random effect for the genomic copy number analysis. Data are expressed as mean ± SE. *Statistically significant differences in change from baseline between treatment groups (p-value < 0.05) as assessed by the linear or generalized linear mixed model.

However, this bifidogenic effect observed at Week 6 did not sustain after 2 weeks of wash-out period (Fig. 3A, Supplementary Fig. S1A). Interestingly, infants in both synbiotic groups had a significantly significantly larger decrease compared to baseline in proportion of *Eubacterium rectale/Clostridium coccoides* than infants in the control group at Week 6 (Syn4: p** < **0.0001; Syn6: p = 0.0002) (Fig. 3C, Supplementary Fig. S1B). The other five taxonomic groups analysed by FISH were not significantly different. Analysis using q-PCR revealed a change from baseline in total bifidobacterial copy numbers at week 6 that was only significantly larger in the Syn4 dose (p = 0.0079) compared to the control product. At week 8, changes in bifidobacterial copy numbers from baseline copy numbers were not significantly different for either dose compared to the control product (Fig. 4E).

Changes from baseline in the *B. breve* M-16V copy numbers at Week 6 and Week 8 were significantly larger in both the Syn4 dose (p < 0.0001, p < 0.0001) and the Syn6 dose (p < 0.0001, p = 0.016) compared to the control product (Fig. 4F and Supplementary Fig. S2). Furthermore, q-PCR analyses showed a significantly higher mean percentage of infants with detectable *B. breve* (prevalence) in the Syn4 group (Fig. 4A) and Syn6 group (Supplementary Fig. S2A) (Syn4: p = 0.0015; Syn6: p = 0.0346) compared to the control group. The mean percentage of infants with detectable *B. breve* M-16V was significantly higher in the Syn4 group (Fig. 4B) and Syn6 group (Supplementary Fig. S2B) at Week 6 (Syn4: p = 0.0002; Syn6: p < 0.0001) and remained significant after the wash-out (Syn4: p = 0.0023; Syn6: p = 0.0256).

q-PCR analysis of potential pathogens demonstrated that the prevalence of infants with detectable *C. difficile* was significantly lower in both Syn4 group (Fig. 4C) and Syn6 group (Supplementary Fig. S2D), closer to the level of the breastfed reference group at Week 6 (Syn4: p = 0.0309; Syn6: p = 0.0006). Interestingly, the prevalence of infants with detectable *C. difficile* remained lower although not significant in the Syn6 group after wash-out (at Week 8) (p = 0.0631) (Supplementary Fig. S2D). The detected *C. difficile* genomic copy numbers were also significantly lower in both Syn4 group (Fig. 4D) and Syn6 group (Supplementary Fig. S2D) after intervention (Syn4: p = 0.0004; Syn6: p = 0.0001). The prevalence of *C. perfringens* tended to be lower in Syn6 group at Week 6 (p = 0.0652).

The prevalence of other pathogens such as *C. jejuni*, EPEC and EAEC was also assessed. *C. jejuni* was below detection level in most infants, whereas EPEC and EAEC were found in low abundances and similar prevalence in each group (data not shown).

### Effect of synbiotics on fecal pH, lactate and short chain fatty acids

**Figure 5:**
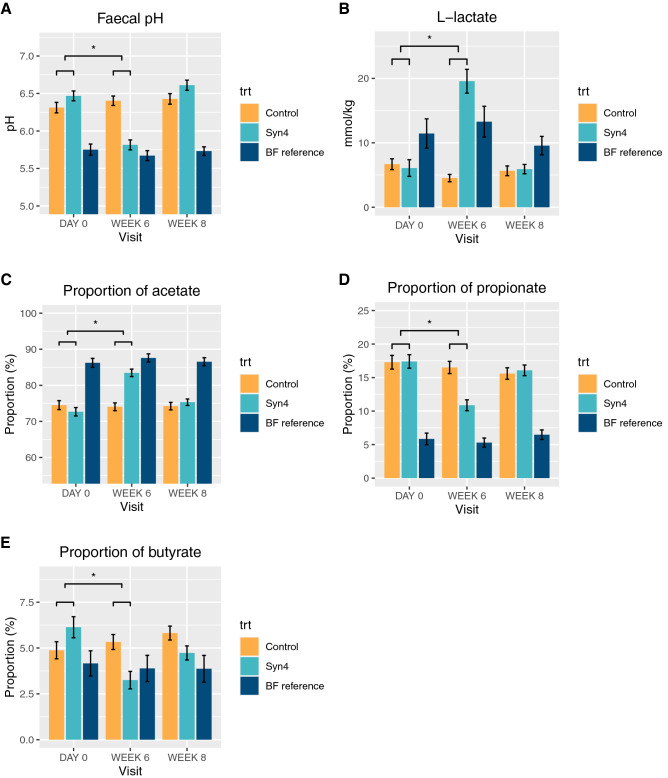
The Syn4 dose resulted in a significantly larger decrease in pH (**A**) and a significantly larger increase in the l-lactate concentration (**B**) and the proportion of acetate (**C**) as compared to control. The proportion of propionate (**D**) and butyrate (**E**) in the Syn4 arm decreased significantly more from baseline as compared to the control arm. Data are expressed as mean ± SE. *Statistically significant differences in change from baseline between treatment groups (p-value < 0.05) as assessed by a longitudinal linear mixed-effect model with intervention, time, study site as fixed factors, intervention by time as interaction term and subject as a random effect.

Compared to the control product, the Syn4 and Syn6 treatments both lead to a larger decrease from baseline in fecal pH (Syn4: p < 0.0001; Syn6: p < 0.0001) and a larger increase from baseline in fecal L-lactate concentrations (Syn4: p < 0.0001; Syn6: p < 0.0001) after 6 weeks of intervention (Fig. 5A,B and Supplementary Fig. S3A,B).

Fecal pH and l-lactate levels in the Syn4 and Syn6 groups were close to (not significantly different from) the breastfed reference. However, the effects did not sustain after the 2 weeks wash-out period. Acetate was the most abundant SCFA detected during the study period in each intervention group (Supplementary Figs. S4 and S5). After 6 weeks of intervention, the increase of acetate (in proportion to propionate and butyrate) from baseline was significantly larger in both Syn4 (Fig. 5C) and Syn6 (Supplementary Fig. S3) groups (Syn4: p < 0.0001; Syn6: p < 0.0001) compared to the control group and closer to the level in the breastfed reference group compared to the control group. The decrease from baseline of propionate proportions (Syn4: p < 0.0001; Syn6: p = 0.0014) and butyrate proportions (Syn4: p < 0.0001; Syn6: p < 0.0015) at Week 6 were significantly larger in both Syn4 (Fig. 5D,E) and Syn6 (Supplementary Fig. S3D,E) groups. Levels of isobutyric and isovaleric acid as well as valeric acid were close or below accurate detection levels throughout the study in all infants (data not shown).

### Redundancy analysis of microbial community composition

**Figure 6:**
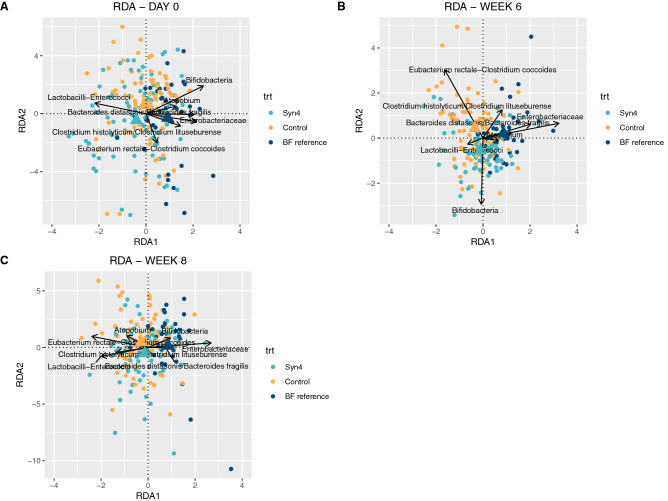
RDA plot for gut microbiota analysis by treatment (control, syn4 and reference) at baseline (**A**), Week 6 (**B**) and Week 8 (**C**). A dot represents each sample and different colors represent different treatment (trt) groups. Triangles indicate centroids of study groups. Statistical significance of differences between groups based on the resulting model was evaluated using an ANOVA like permutation test.

Results based on RDA showed that after 6 weeks of intervention, Sy4 (Fig. 6) and Syn6 groups (Supplementary Fig. S6) shifted away from the control group, suggesting that the use of synbiotics influences the microbial community composition. Using pairwise permutation tests, the gut microbiota composition for both the Syn4 and Syn6 groups were found to be significantly different from the control group (Syn4: p = 0.002; Syn6: p = 0.002). Subjects with increasing proportions of bifidobacteria showed a decrease in proportion of *Eubacterium rectale–Clostridium coccoides* after 6 weeks of intervention (and vice-versa) (Figs. 3C and 6B).

### Stool characteristics and adverse events

Stool frequency did not differ among the three intervention groups throughout the study. However, stool consistency was significantly softer in both synbiotic groups by the end of the intervention period (Supplementary Figs. S7 and S8) but not after the wash-out period.

No serious adverse events were recorded during the study. All formulas were well tolerated and all groups showed a comparable safety profile based on the number and severity of adverse events. The percentage of infants experiencing adverse events was similar in the three intervention groups. All infants grew well according to WHO Child Growth Standards.

## Discussion

In this study, we have shown that a specific synbiotic mixture (*B. breve* M-16V and scGOS/lcFOS (9:1)) at two different doses increased the bifidobacteria proportion in healthy infants. This helps infants to acquire infant-type *Bifidobacterium* species, and enrich bifidobacteria abundance. This transient increase in amounts of infant-type bifidobacteria steers the infant’s gut microbiota towards a stable ecosystem, which further benefits gut maturation and immune development during early life^31–33^.

In addition to the enhancement of the total bifidobacteria proportion, this unique synbiotic increased the prevalence of *B. breve* and *B. breve* M-16V in the infants’ gut. It is interesting to note that these effects sustained after 2 weeks wash-out period. In a recent study performed in C-section born infants with this same specific synbiotic combination, *B. breve* M-16V was detected in more than 40% of the infants after a 6 weeks follow-up period^25^. In addition to an increased prevalence of *B. breve,* this intervention also resulted in an increase of *B. bifidum* and *B. longum,* but had no effect on *B. catenulatum*^25^. In another clinical trial comparing the impact of *B. infantis* (infant-type) and *B. lactis* (non-infant-type) on gut microbiota colonization in premature infants, *B. infantis* was shown to be more effective at colonizing than *B. lactis* in both formula-fed and human milk-fed premature infants^34^. These findings suggest that infant-type of *Bifidobacterium* species can survive and colonize an infant’s gut better than non-infant-type species. More studies on the colonization potential and health benefits of infant-type bifidobacterial strains are needed to provide a better guidance in probiotic *Bifidobacterium* selection for early life.

In agreement with other studies^26^, supplementation with *B. breve* M-16V and scGOS/lcFOS (9:1) decreased the proportion of *E. rectale–C. coccoides*; a broad group of bacteria capable of producing butyrate and secondary bile acids. Though secondary bile acids were not measured in this study, this finding is consistent with low butyrate profiles in infants supplemented with synbiotics. Levels of butyrate are very low in breastfed infants before weaning^35^. It has recently been hypothesized that the butyrate production stage is critical for infant gut maturation and may be associated with health outcomes such as allergy^36^. A longer clinical study or a follow up might elucidate the relationship between butyrate production and health outcomes in later life. Changes in gut microbiota composition were in line with changes in the gut microbiota metabolic activity in both synbiotic groups. Supplementation with *B. breve* M-16V and scGOS/lcFOS (9:1) promoted an acidic environment by increasing the production of acetate and lactate, resembling the gut environment of healthy breastfed infants. Constipation or hard stools are more common among formula-fed infants than breastfed infants (9.2% in formula-fed infants vs 1.1% in breastfed infants)^37,38^. In our study, the intervention with this specific synbiotic mixture resulted in softening of the stool. Although the number of infants experienced constipation or hard stool in this study was generally low, taking all the above findings, we hypothesize that this specific synbiotic mixture could reduce hard stools/constipation episodes in formula-fed infants.

During early life, the gut microbiota is constantly exposed to environmental challenges such as antibiotics treatment and formula feeding, which have been shown to influence *C. difficile* levels as well as the abundance of other opportunistic pathogens. Establishment and maintenance of a healthy microbial community will increase the gut homeostasis and hence may increase the gut microbiota resilience. In this study, *B. breve* M-16V and scGOS/lcFOS (9:1) supplementation significantly reduced *C. diffficile* levels closer to what is observed in breastfed infants. This effect of synbiotics on *C. difficile* reduction has not been demonstrated in other clinical studies before. An in vitro study using co-culture methods showed that *B. breve* or *B. longum* combined with scFOS reduced *C. difficile* growth and toxicity, whereas an opposite effect was observed for *B. animalis* subsp. *lactis* Bb12^39^. This confirms that not all probiotic *Bifidobacterium* species have comparable effects on pathogen reduction. Reduction of potential pathogens may be a key step towards reducing infections in early life. Reduction of *C. diffficile* abundance as well as the reduction trend of *C. perfringens,* EPEC and EAEC, suggests that this specific synbiotic mixture may be able to protect infants against *C. difficile* infections and other gastrointestinal infectious diseases during early life. The facts that this specific synbiotic mixture reduced potential pathogens and increased bifidobacteria proportions as well as the prevalence of *B. breve* implies that this synbiotic mixture creates a homeostatic beneficial microbial community and thus improves gut microbiota resilience. The resilience of a healthy microbiota further protects infants from dysbiosis-related diseases such as allergy and infections^40^.

Interestingly, it was observed that this synbiotic mixture with a probiotic dose close to the level of bacteria found in human milk, which is about 10^4^ cfu/ml (daily intake about 10^7^ cfu), is sufficient to influence the healthy infants’ gut microbiota and create a gut environment closer to breastfed infants. A previous study using different doses of probiotic *B. lactis* in C-section born infants from birth till 12 months, showed that formula containing 10^4^ cfu/g or 10^7^ cfu/g *B. lactis* or breast milk provided similar effects (diarrhoea, immune and gut maturation and total bifidobacteria counts) at 12 months^41^. However, no control formula was tested. Also, subjects in the breastfed reference group (recommended for a minimum of 4 months) were mixed-fed with a formula without any probiotics supplementation for up to 12 months. These factors complicate the conclusions drawn by Aglatzi et al.^41^ on the comparison between probiotics supplementation and breastfeeding.

A limitation of this study is the fact that subjects were investigated over a limited period of 8 weeks. Prospective studies including large numbers of inclusions over longer time spans are warranted to assess the long term effect of early life synbiotics administration and gut microbiota development and health consequences later in life. More clinical studies in infants are needed to further evaluate the effects of different doses of synbiotics on the infants’ gut microbiota development and subsequent clinical health outcomes including long-term health.

## Conclusion

In healthy infants, a synbiotic mixture of an infant-type *Bifidobacterium*, *B. breve* M-16V combined with scGOS/lcFOS (9:1) at a level closer to the bacterial levels in human milk, created a gut environment closer to the breastfed reference group. Supplementation of this specific synbiotic mixture helps infants develop a preferred gut environment and support gut microbiota resilience by increasing bifidobacteria proportions and decreasing *C. difficile*. Further multicentered randomized double-blind controlled studies conducted with different doses of synbiotics and strain-specific probiotics are needed to further understand their impact on health outcomes of infants, such as infections and allergies.

## Supplementary Information

Supplementary Information 1.

Supplementary Information 2.

## Abbreviations

EPEC: Enteropathogenic *Escherichia coli*
EAEC: Enteroaggregative *Escherichia coli*
HMOs: Human milk oligosaccharides
scGOS/lcFOS: Short-chain galacto-oligosaccharides and long-chain fructo-oligosaccharides
CFU: Colony forming unit
Syn4: Control formula supplemented with 0.8 g/100 ml scGOS/lcFOS and *B. breve* M-16V at a dose of 1 × 10^4^ cfu/ml
Syn6: Control formula supplemented with 0.8 g/100 ml scGOS/lcFOS and *B. breve* M-16V at a dose of 1 × 10^6^ cfu/ml
FISH: Fluorescent in situ hybridization
SCFA: Short chain fatty acid
ITT: Intention-to-treat
AST: All-subjects-treated
MMRM: Mixed-effect model for repeated measures
ANCOVA: Analysis of covariance
GLMM: Generalized linear mixed model
RDA: Redundancy analysis
TRT: Treatment
WHO: World Health Organization
