# Supplementary material for: Impact of synbiotics on gut microbiota during early life: a randomized, double-blind study
Source: Sci Rep. 2021 Feb 11;11:3534. doi: 10.1038/s41598-021-83009-2 (PMC7878856; doi:10.1038/s41598-021-83009-2)
Supplement: Supplementary file 1 — Supplementary Information 1. [file 41598_2021_83009_MOESM1_ESM.pdf]

Impact of synbiotics on gut microbiota during early life: a randomized, double-blind study

Nopaorn Phavichitr MD [1], Shugui Wang, PhD [3], Sirinuch Chomto, MD, PhD[4], Ruangvith Tantibhaedhyangkul, MD[1], Alexia Kakourou, PhD[2], Sukkrawan Intarakhao, MD[5], Sungkom, Jongpiputvanich, MD[5], COLOR study group[*] , Guus Roeselers, PhD[2], Jan Knol, PhD[2,6]

SUPPLEMENTAL INFORMATION & METHODS

TRIAL REGISTRATION & ETHICAL APPROVAL

Participating centres obtained approval from their independent local Ethical Review Boards (Institutional Review Board Chulalongkorn University, Bangkok; Institutional Review Board, Royal Thai Army Medical Department, Bangkok; Human Research Ethics Committee, Thammasat University, Pathum Thani, Thailand). Written informed consent was obtained from all parents before inclusion in the study. The study was registered in ClinicalTrials.gov (March 18, 2013; #NCT01813175).

The study was conducted according to ICH-GCP principles, and in compliance with the principles of the 'Declaration of Helsinki' (59th WMA General Assembly, Seoul, October 2008) and with the local laws and regulations of the country where the study was performed.

RANDOMISATION & UNBLINDING

Based on the order in which they enter the study, subjects were assigned a randomisation number (whereby stratification is applied for site). After assignment of a randomisation number the correspondingly numbered randomisation envelope were opened, which contained the code of the study product that the subject will receive (A, B, C, D, E or F). The product codes were assigned using a computer random number generator. The details of the randomisation remained unknown to the investigator, to the site staff, and to the study staff from Danone - Nutricia Research (except for the statistician who was responsible for generating the randomisation sequence, and the Supplies Manager who needed to be unblinded in order to label the study products, to create unblinding envelopes, etc.).

At each study site one sealed master unblinding envelope was available, which contained a sealed unblinding envelope for each of the treatment codes. Only in case of a medical emergency that required unblinding of the study product, the investigator was authorized to open the envelope for the specific study product code. The unblinding information should only be shared with those who need the information to treat the subject properly.

SAMPLE SIZE CALCULATION

Sample size was calculated was based on the following estimates:

1. Effect on intervention: there was no change in percentage of *Bifidobacterium* sp. in the faeces of infants in the group using infant formula containing synbiotics and a decrease of 17% in the control group after 1 week of intervention (based on previous Nutricia Research study, SYNBAD; https://www.trialregister.nl/trial/97)
2. Standard deviation (SD) of difference: 23% (based on previous Danone Nutricia Research study; SYNBAD; https://www.trialregister.nl/trial/97).

Using the above-mentioned a-priori estimates, with a two-sided significance level () of 0.05, and a power of 80%, a sample size of 30 each group is assumed to be sufficient to detect a statistically significant difference as calculated by SAS statistical software. Assuming a 30% drop-out rate, the number of subjects needed per group is 43.

For the study with the groups Breastfeeding, Investigational Formula I and II and the Control-Formula the total number of subjects needed is 4 x 43 = 172. However, after conducting the First Interim Analysis, the Independent Committee suggested to increase the sample size by increasing the number of study subjects for the Control, Investigational Product I and II intervention groups, from the original 43 to 50 subjects per group. The Breastfeeding group will remain the same at 43 subjects.

The assumption for sample size calculation was further checked in the Second Interim Analysis, and the recommendations by the Independent Committee are as follows: Based on the change from baseline in proportion of *Bifidobacterium* sp., it was estimated that 60 evaluable subjects per arm are needed to demonstrate 80% chance of detecting a significant difference between the study arms showing the biggest difference. To demonstrate 80% chance of showing a significant difference between the groups with the second larger difference, 77 evaluable subjects would be required. Of the two scenarios, the sample size required for the study is 77 evaluable subjects per intervention group. As the observed drop-out rate (<5%) is much lower than the original assumption of 30%, a dropout rate of 5% is now taken as the assumption for sample size calculation. Therefore, 82 subjects per arm should be included in the intervention groups. The drop-out rate was closely monitored during the study. For the Breastfeeding Group, the number of subjects in would not be increased, and would remain at 43 subjects. Based on these calculations 289 subjects should be included in the entire study.

A total of 290 subjects were recruited, of whom 247 subjects were randomized into three intervention groups and 239 subjects completed the study. The other 43 subjects were included in the non-randomized breast-fed reference group and 42 subjects completed the study (See Fig.2 in main manuscript).

STUDY PRODUCT

Name and description of study products

Run-in product:

Regular non-hydrolysed cow's milk based infant formula

Control product:

Regular non-hydrolysed cow's milk based infant formula

Wash-out product:

Regular non-hydrolised cow's milk based infant formula

Investigational product I (Syn6):

Regular non-hydrolysed cow's milk based infant formula with added synbiotic mixture of galactooligosaccharides and fructo-oligosaccharides (scGOS/lcFOS; 0.8 g/100ml, ratio 9:1) and 1.0 x 10^6 CFU of B. breve M-16V/ml.

Investigational product II (Syn4):

Regular non-hydrolysed cow's milk based infant formula with added synbiotic mixture of galactooligosaccharides and fructo-oligosaccharides (scGOS/lcFOS; 0.8g/100 ml, ratio 9:1) and 1.0 x 10^4 CFU of B. breve M-16V/ml.

All study products are enriched with prepared product of LCPUFA, comprising AA and DHA (ratio 0.35:0.32 per 100g fatty acid). All study products were provided by Danone Nutricia Research.

Summary of known and potential risks and precautions

The study product is not to be used in infants with established or expected cows' milk allergy, soy protein allergy, fish protein allergy, lactose intolerance, fiber free diet or galactosaemia. No adverse events are expected due to the administration of the investigational products or control product. In some studies, it has been demonstrated that use of synbiotics is associated with softer stools (a consistency closer to that of breastfed infants' stools).

Preparation, packaging, labelling and storage

Preparation and directions for use:

The study product is prepared and administered according to the instructions enclosed with the formula. The prepared formula should be consumed within 2 hours of preparation.

Packaging and labelling;

The infant milk formula is supplied in 600 g pouch. Boxes are labelled in accordance with applicable laws and regulations in such a way that the double-blind design of the study is effectively maintained throughout the study. Labels on the investigational product as well as the control product contain information required for regulatory as well as identification purposes.

Storage conditions:

The investigator is responsible that the study product is stored in a secure, limited access storage area at an ambient stable, preferably cool and dry place (between 2oC and 25oC) and protected from extremes of light and humidity.

The subjects' parents are asked to keep the study product stored in a secure, cool dry place (not in a refrigerator) in their house, protected from direct sunlight. After opening the pouch, it should be stored in a clean, dry and airtight container.

FECAL SAMPLES ANALYSES

FISH analyses of the abundance of 7 main faecal microbiota taxa

All 16S rRNA-targeted oligonucleotide probes, their sequence and their targeted bacterial groups are listed in Table 1 below. All probes were purchased from MWG (Ebersgreg, Germany) and were covalently linked at their 5'-end with Cy3. The Nucleic acid stain DAPI (Invitrogen, Leiden, the Netherlands) was used for determining total fecal cell counts. PFA-fixed fecal samples were hybridized with the specific probes and then counted using an automated Olympus AX70 epifluorescence microscope equipped with a Lang LStep13 8 slides-stage (Paes Nederland BV, Zoeterwoude, the Netherlands) and an F-View II charge-coupled device (Soft Imaging System GmbH, Münster, Germany) and image analysis software. The percentage of bacterial cells was determined at 25 randomly chosen positions on each well by counting all cells using a DAPI filter set (SP100; Chroma Technology Corp., Brattleboro, VT, USA) and by counting the targeted bacterial group using a Cy3 filter set (41007; Chroma Technology Corp.).

q-PCR quantification

The absolute gene counts of total *Bifidobacterium* species and the probiotic strain *B. breve* M-16V and the potential pathogens *Clostridium jejuni*, *Clostridium difficile*, *Clostridium perfringens*, *Staphilococcus aureus*, *Escherichia coli* EAEC, *Escherichia coli* EPEC were assessed with quantitative real-time PCR (Q-PCR). Two Q-PCR chemistries (SYBR Green and TaqMan) were selected and carried out using ABI Prism 7900HT (Applied Biosystems, California, USA). SYBR Green PCR master mix (Applied Biosystems, Carlsbad, CA) and TaqMan universal master mix (Applied Biosystems, Austin, USA) were used for their respective assays. Primers were selected and/ or designed to amplify specific regions of the 16S rRNA gene or the 16S-23S intergenic spacer region or specific genomic regions with their respective optimized Q-PCR condition (see Table 2 below). Standards for each target were generated from their respective genomic DNA by endpoint PCR with their respective primer pairs. The specificity of the amplified PCR product was checked using gel electrophoresis (1.5% agarose + 1x TAE buffer) prior to purification using MinElute PCR Purification Kit (QIAGEN) as per manufacturer protocol. The concentrations of the purified amplicons were

measured using NanoDrop 2000 (ThermoScientific, DE, USA) and the copy number/µl were calculated (DNA concentration / (fragment length x weight of base pair)) before diluting to a range of 10^1-10^6 copies/µl of standards used in each assay. In brief, fecal genomic DNA was diluted 100, 1,000 and 10,000 times and dispensed into 384-wells optical plate using Microlab Nimbus robotics (Hamilton, Ne Vada USA). Q-PCR conditions were 1 cycle of 95°C for 20s followed by amplification at 95°C for 1s, 60°C for 20s for 40 cycles and 1 cycle of 95°C for 15s, 60°C for 15s and 95°C for 15s with readings collected at the last step for melting curve analysis in SYBR Green assays. For TaqMan assays, the conditions were set at 1 cycle of 50°C for 2 minutes, 1 cycle of 95°C for 10 minutes, followed by 40 cycles of amplification at 95°C for 15s and 60°C for 1 minute. SDS 2.4 (Applied Biosystems, USA) was used to visualize and check abnormality of curves deviating from standard amplification. Raw data were then exported into Excel, where the Ct values were recalculated for log Copy numbers/g faeces using in-house optimized macros. The final results were then used for statistical analysis.

Table 1: 16S rRNA-gene targeted oligonucleotide probes used for FISH

	**Sequence from 5' to 3'-end**	**Targeted bacterial groups**	**References**
Ato291	GGTCGGTCTCTCAACCC	*Atopobium* group	[1]
Bdis656	CCGCCTGCCTCAAACATA	*Bacteroides distasonis* group	[2]
Bfra602	GAGCCGCAAACTTTCACAA	*Bacteroides fragilis* group	[2]
Bif164-mod	CATCCGGYATTACCACCC	*Bifidobacterium* group	[3]
Chis150	TTATGCGGTATTAATCTYCCTTT	*Clostridium histolyticum* group	[2]
Clit135	GTTATCCGTGTGTACAGG	*Clostridium lituseburense* group	[2]
Ec1531	CACCGTAGTGCCTCGTCATCA	Enterobacteriaceae (*E.coli*, *Shigella*, *Salmonella*, *Klebsiella*)	[4]
Erec482	GCTTCTTAGTCARGTACCG	*Eubacterium rectale–Clostridium coccoides* cluster	[2]
Lab158	GGTATTAGCAYCTGTTTCCA	*Lactobacillus–Enterococcus* group	[5]

Table 2: 16S rRNA gene, 16S-23S intergenic spacer region or specific genomic region targeted primers used for Q-PCR analysis

Target	Primer Label	Sequence (5' to 3')	Amplicon length (bp)	Probe (if applicable)	Ref
**Total Bacteria**	338F	ACT CCT ACG GGA GGC AGC	178	SYBR	This study
	533R	TTA CCG CGG CTG CTG GCA C			
**Total *Bifidobacterium***	F-bifido	CGC GTC YGG TGT GAA AG	244	FAM-NFQ-MGB	6
	R-bifido	CCC CAC ATC CAG CAT CCA			
	MGB-bifido	AAC AGG ATT AGA TAC CC			
B. breve	F_breve_IS	GTG GTG GCT TGA GAA CTG GAT AG	118	FAM-NFQ-MGB	7
	R_breve_IS	CAA AAC GAT CGA AAC AAA CAC TAA A			
	P_breve_IS	TGA TTC CTC GTT CTT GCT GT			
***B. breve* M-16V**	JLa1-F	GGC CAC CAG TAT GGT CTT ATC C	63	FAM-NFQ-MGB	This study
	JLa1-R	TCG TGC CAT TCG CTA TTG C			
	JLa1-P	CTT GGG CGC CAT GAT			
Campylobacter jejuni	16S-UNI-CampyF1	CACGTGCTACAATGGCATATACAA	78	FAM-NFQ-MGB	8
	16S-UNI-CampyR1	CCGAACTGGGACATATTTTATAGATTT			
	16S-UNI-CampyP1	AGACGCAATACCGTGAGGT			
Clostridium difficile	16S-Cldif -F	GCAACGCGAAGAACCTTACCTA	67	VIC-MGB	9
	16S-Cldif -R	GAAGGGAACTCTCCGATTAAGGA			
	16S-Cldif -P	TGACATCCCAATGACA			
Clostridium perfringens	16S-Clperf -F	GAACCTTACCTACACTTGAC	76	VIC-MGB	9
	16S-Clperf -R	CCACCTGTCACCTTGTCC			
	16S-Clperf -P	TGCATTACTCTTAATCGAG			
Staphilococcus aureus	16S-S.aur-F1	GCG AAG AAC CTT ACC AAA TCT TG	87	FAM-NFQ-MGB	10
	16S-S.aur-R1	TGC ACC ACC TGT CAC TTT GTC			
	6S-S.aur	CAT CCT TTG ACA ACT CT			
***Escherichia coli* EAEC**	pCVD432 EA-1-F	AGG TTT GAT ATT GAT GTC CTT GAG GA	152	FAM-TAMRA	11
	pCVD432 EA-2 -R	TCA GCT AAT AAT GTA TAG AAA TCC GCT GTT			
	pCVD432 EA-S-Probe	CAT GTT CCT GAG AGT GCA ATC CCA GAC ATT AC			
***Escherichia coli* EPEC**	EPEC-EAE-F	CAT TGA TCA GGA TTT TTC TGG TGA TA	102	VIC-MGB	11
	EPEC-EAE-R	CTC ATG CGG AAA TAG CCG TTA			
	EPEC-EAE-Probe	ATA GTC TCG CCA GTA TTC GCC ACC AAT ACC			

Measurements of fecal pH and selected microbial metabolites

SCFA content (acetic, propionic, n-butyric, iso-butyric, n-valeric and iso-valeric acids) in faecal samples were quantitatively determined by a Varian 3800 gas chromatograph (GC) (Varian Inc., Walnut Creek, CA, USA) equipped with a flame ionization detector. Lactate was measured enzymatically using a D-lactic acid/ L-lactic acid detection kit containing D- and L-lactate dehydrogenase (Boehringer Mannheim, Mannheim, Germany). Fecal samples were thawed and pH was measured directly at room temperature using a Handylab pH meter (Schott Glas,Mainz, Germany) equipped with an Inlab 423 pH electrode (Mettler-Toledo, Columbo, Schwerzenbach, Switzerland).

REFERENCES

1. Harmsen HJ, Wildeboer-Veloo AC, Grijpstra J, Knol J, Degener JE, Welling GW. Development of 16S rRNA-based probes for the Coriobacterium group and the Atopobium cluster and their application for enumeration of Coriobacteriaceae in human feces from volunteers of different age groups. *Appl Environ Microbiol.* 2000;66:4523-4527.
2. Franks AH, Harmsen HJ, Raangs GC, Jansen GJ, Schut F, Welling GW. Variations of bacterial populations in human feces measured by fluorescent in situ hybridization with group-specific 16S rRNA-targeted oligonucleotide probes. *Appl Environ Microbiol.* 1998;64:3336-3345.
3. Langendijk PS, Schut F, Jansen GJ, et al. Quantitative fluorescence in situ hybridization of Bifidobacterium spp. with genus-specific 16S rRNA-targeted probes and its application in fecal samples. *Appl Environ Microbiol.* 1995;61:3069-3075.
4. Poulsen LK, Licht TR, Rang C, Krogfelt KA, Molin S. Physiological state of Escherichia coli BJ4 growing in the large intestines of streptomycin-treated mice. *J Bacteriol.* 1995;177:5840-5845.
5. Hermie J. M. Harmsen PEFSGWW. A 16S rRNA-targeted Probe for Detection of Lactobacilli and Enterococci in Faecal Samples by Fluorescent In Situ Hybridization. *Microbial Ecology in Health and Disease.* 1999;11:3-12.

6. Delroisse J-M, Boulvin A-L, Parmentier I, Dauphin RD, Vandenbol M, Portetelle D. Quantification of Bifidobacterium spp. and Lactobacillus spp. in rat fecal samples by real-time PCR. *Microbiol Research.* 2008;163:663-670.
7. Haarman M, Knol J. Quantitative real-time PCR assays to identify and quantify fecal Bifidobacterium species in infants receiving a prebiotic infant formula. *Appl Environ Microbiol.* 2005;71:2318-2324.
8. de Boer P, Rahaoui H, Leer RJ, Montijn RC, van der Vossen JM. Real-time PCR detection of Campylobacter spp.: A comparison to classic culturing and enrichment. *Food Microbiol*. 2015; 51:96-100.
9. Jurburg SD, Cornelissen JJBWJ, de Boer P, Smits MA, Rebel JMJ. Successional Dynamics in the Gut Microbiome Determine the Success of *Clostridium difficile* Infection in Adult Pig Models. *Front Cell Infect Microbiol.* 2019;9:271.
10. Chang W, Small DA, Toghrol F, Bentley WE. Global transcriptome analysis of Staphylococcus aureus response to hydrogen peroxide. *J Bacteriol*. 2006;188(4):1648-1659.
11. Hardegen C, Messler S, Henrich B, Pfeffer K, Würthner J, MacKenzie CR. A set of novel multiplex Taqman real-time PCRs for the detection of diarrhoeagenic Escherichia coli and its use in determining the prevalence of EPEC and EAEC in a university hospital. *Ann Clin Microbiol Antimicrob*. 2010;9:5.
