# Supplementary material for: Impact of synbiotics on gut microbiota during early life: a randomized, double-blind study
Source: Sci Rep. 2021 Feb 11;11:3534. doi: 10.1038/s41598-021-83009-2 (PMC7878856; doi:10.1038/s41598-021-83009-2)
Supplement: Supplementary file 2 — Supplementary Information 2. [file 41598_2021_83009_MOESM2_ESM.pdf]

Impact of synbiotics on gut microbiota during early life: a randomized, double-blind study

Nopaorn Phavichitr MD [1], Shugui Wang, PhD [3], Sirinuch Chomto, MD, PhD[4], Ruangvith Tantibhaedhyangkul, MD[1], Alexia Kakourou, PhD[2], Sukkrawan Intarakhao, MD[5], Sungkom, Jongpiputvanich, MD[5], COLOR study group[*], Guus Roeselers, PhD[2], Jan Knol, PhD[2,6]

Supplemental Figures & Tables

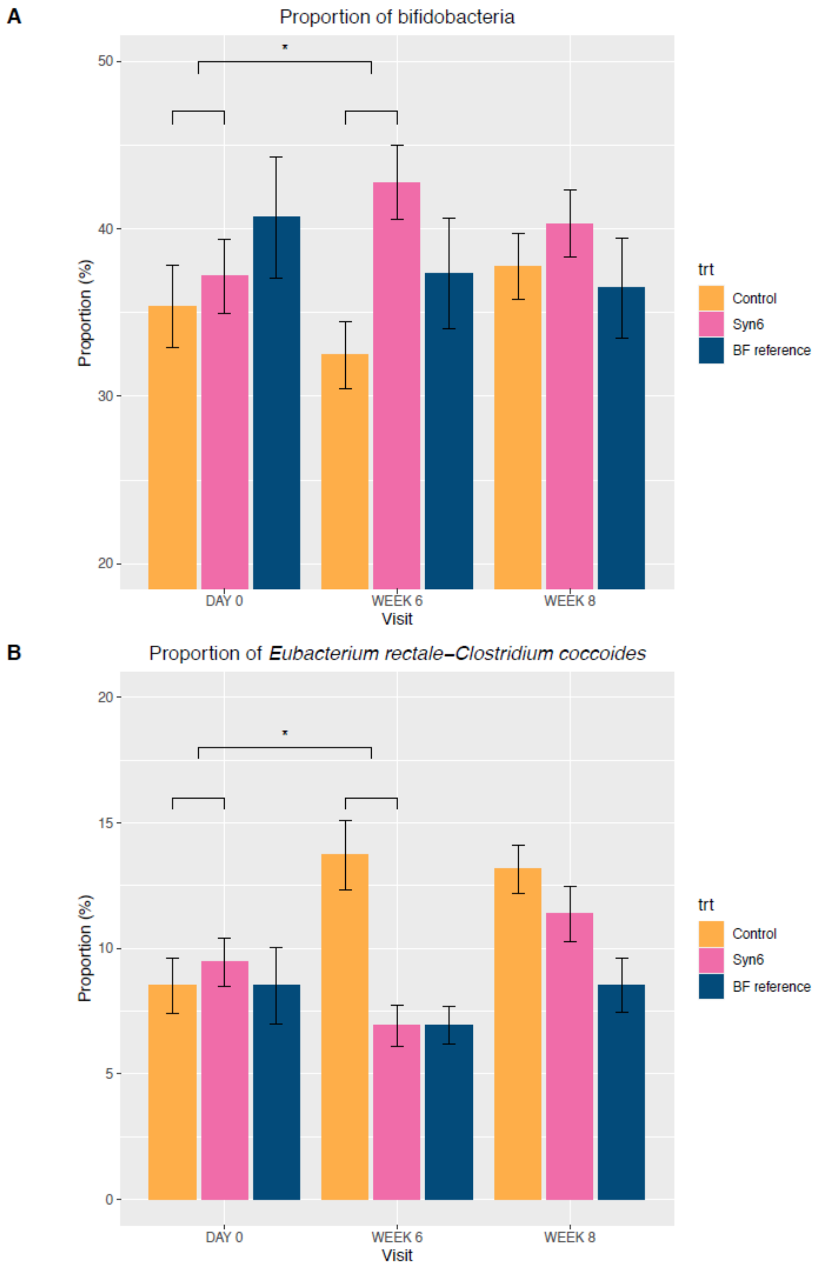

Supplemental Fig.1. FISH analyses showed that the Syn6 dose treatment (trt) increased the proportion of bifidobacteria (A). (The proportion of *Eubacterium rectale-Clostridium coccoides* decreased from baseline compared to control (B). A longitudinal linear mixed model was used with intervention, time, study site as fixed factors, intervention by time as interaction term and subject as a random effect. trt=treatment. Data is expressed as mean ± SE. * p-value < 0.05.

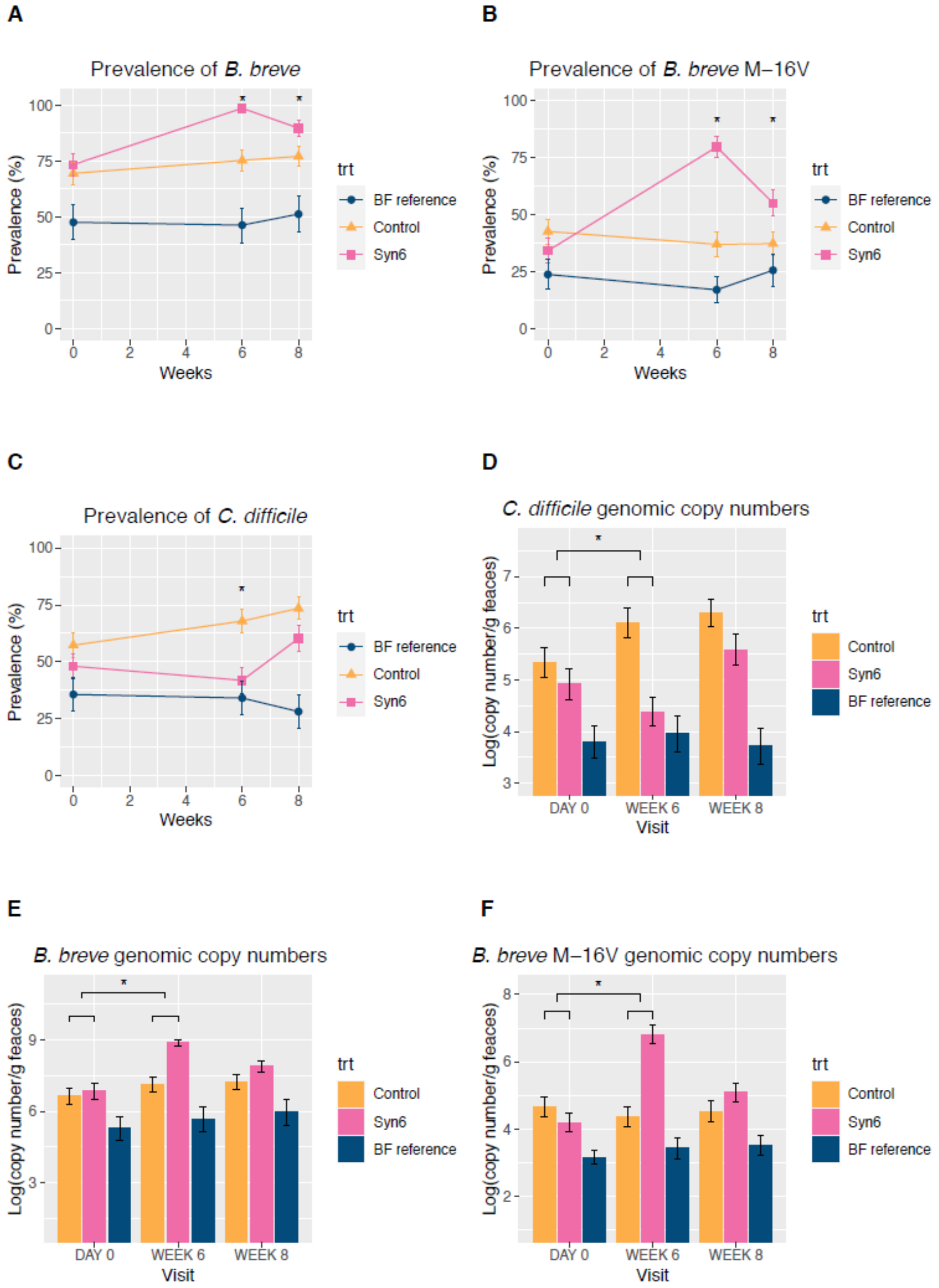

Supplemental Fig.2. q-PCR analyses showed that the Syn6 doses increased the prevalence of *B. breve* (A) and *B. breve* M-16V (B) and decreased *C. difficile* prevalence (D). Detected *C. difficile* genomic copy numbers were significantly lower in the Syn6 group (C). Prevalence of infants with detectable *C. difficile* was significantly lower in the Syn6 group, closer to the level of the breast-fed reference group at Week 6 (p=0.0006) (D). Prevalence of infants with detectable *C. difficile* remained lower although not significant in the Syn6 group after wash-out (at Week 8) (p=0.0631)(D). The increase in total amount of bifidobacterial copy numbers from baseline was significantly larger (p=0.0004) at Week 6 in the Syn6 group compared to control (E). Increase in the *B. breve* M-16V copy numbers from baseline was significantly larger in the Syn6 group compared to control at Week 6 (p<0.0001) (F). A generalized linear mixed model (GLMM) was used with intervention, time, study site as fixed factors, intervention by time as interaction term and subject as a random effect for the analysis of the binary transformed (detected/non-detected) data and the estimation of prevalence of detected values. A longitudinal linear mixed-effect model was used with intervention, time, study site as fixed factors, intervention by time as interaction term and subject as a random effect for the genomic copy number analysis. Data are expressed as mean ± SE. * indicates statistically significant differences in change from baseline between treatment groups (p-value < 0.05) as assessed by the linear or generalized linear mixed model.

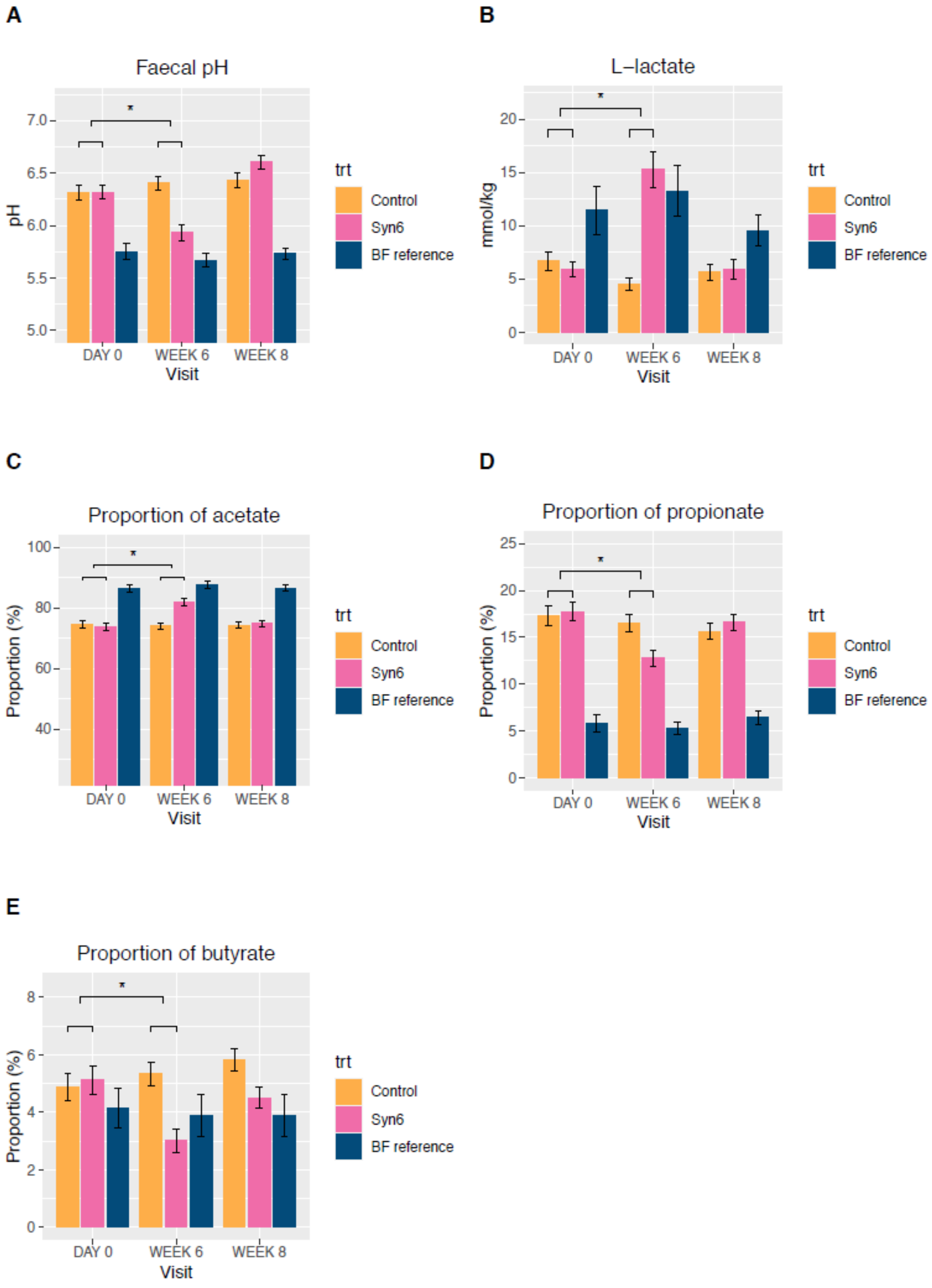

Supplemental Fig.3. The Syn6 dose resulted in a significantly larger decrease in pH (A) and a significantly larger increase in the L-lactate concentration (B) and the proportion of acetate (C) as compared to control. The proportion of propionate (D) and butyrate (E) in the Syn4 arm decreased significantly more from baseline as compared to the control arm. Data are expressed as mean ± SE. * indicates statistically significant differences in change from baseline between treatment groups (p-value < 0.05) as assessed by a longitudinal linear mixed-effect model with intervention, time, study site as fixed factors, intervention by time as interaction term and subject as a random effect.

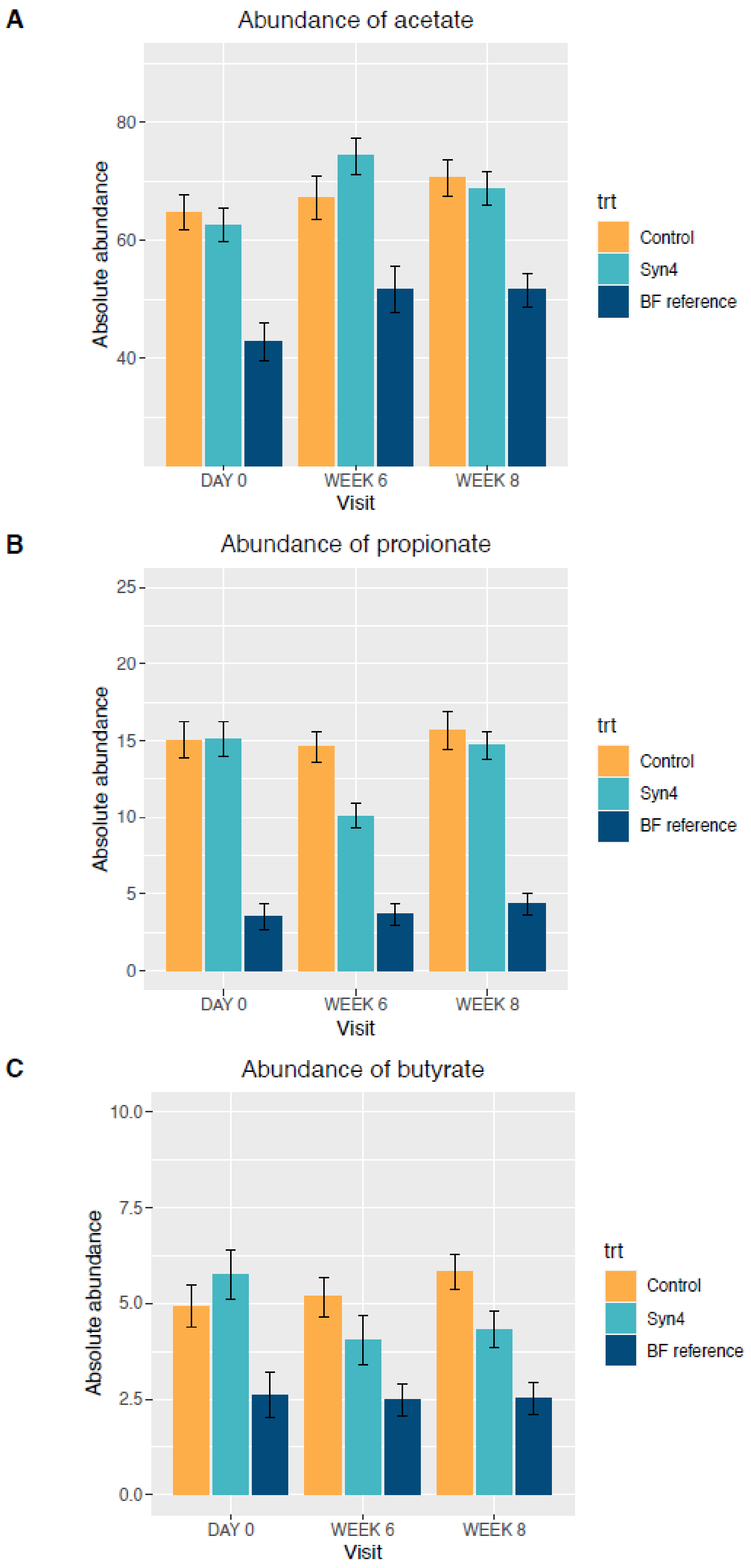

Supplemental Fig.4. Absolute concentrations (mmol/kg) of acetate (A), propionate (B) and butyrate (C) measured in fecal samples from the Syn4 group.

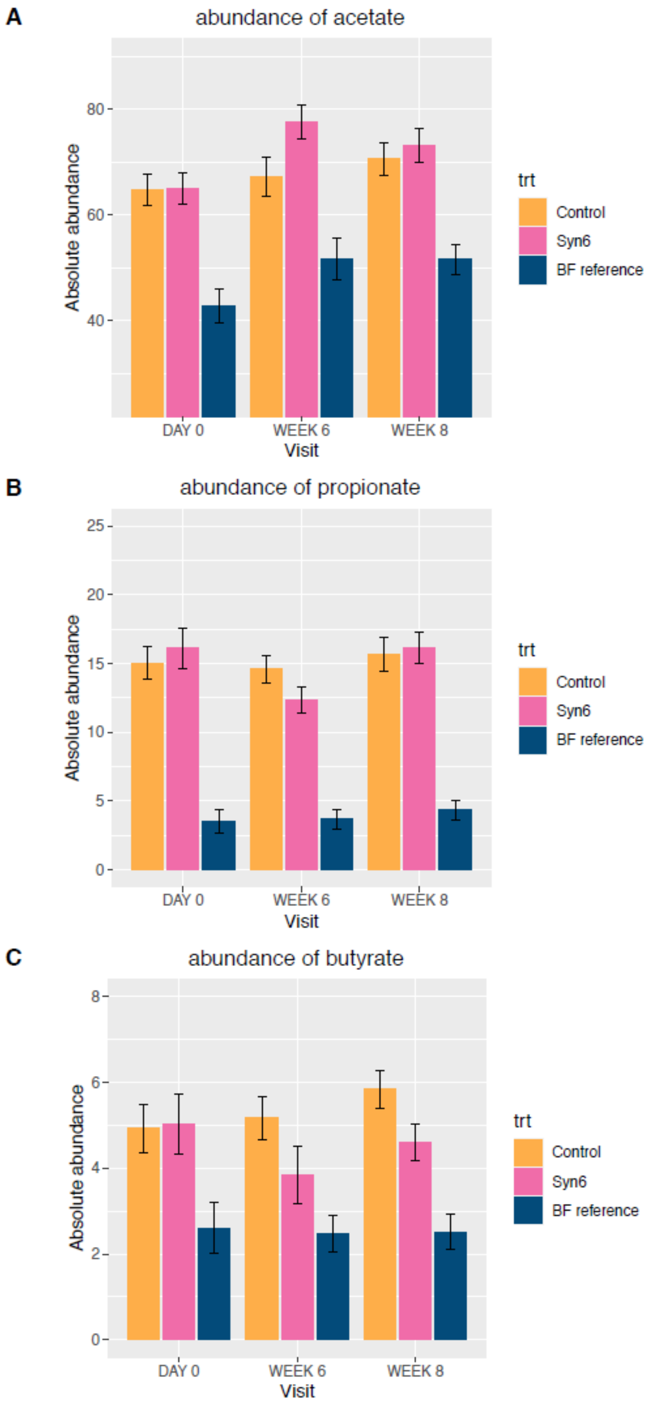

Supplemental Fig.5. Absolute concentrations (mmol/kg) of acetate (A), propionate (B) and butyrate (C) measured in fecal samples from the Syn6 group.

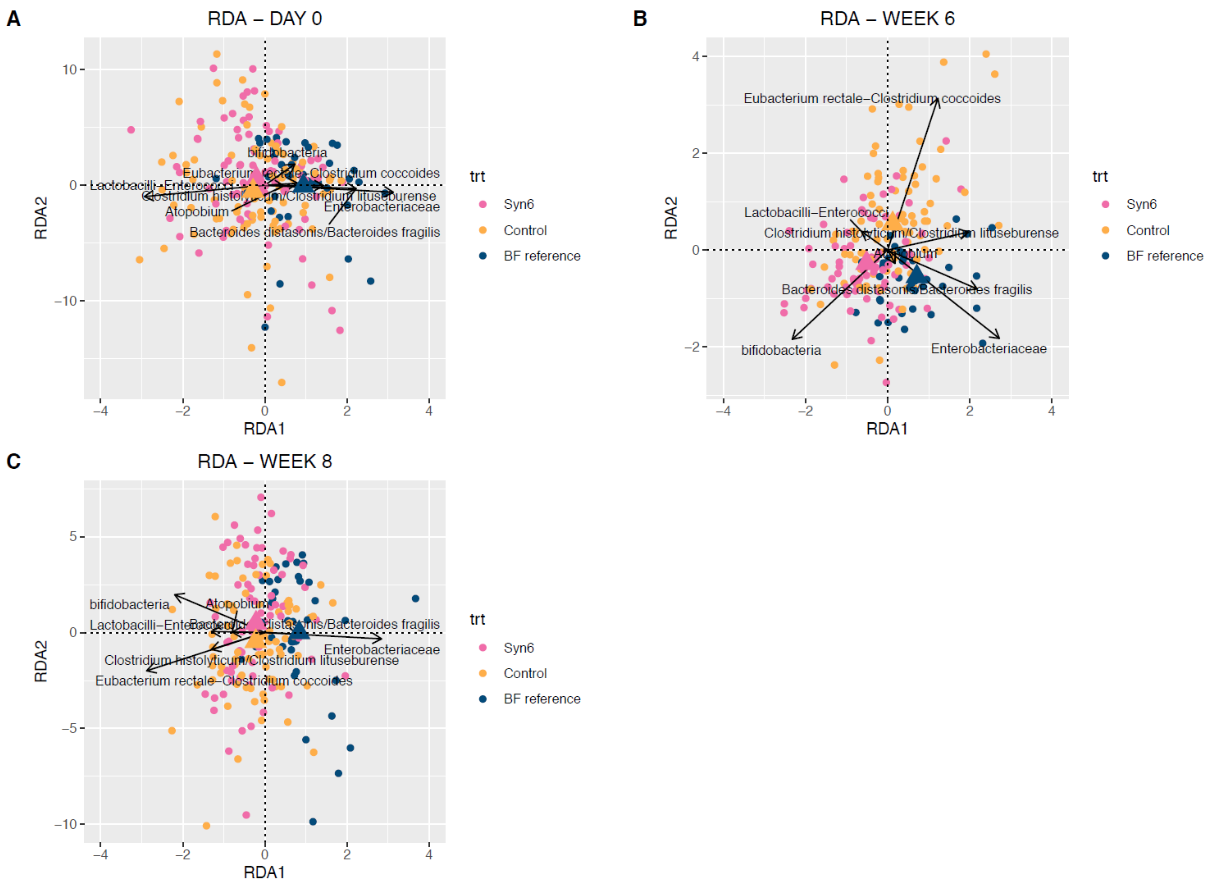

Supplemental Fig.6. RDA plot for gut microbiota analysis by treatment (control, syn6 and reference) at baseline (A), Week 6 (B) and Week 8 (C). A dot represents each sample and different colors represent different groups. Triangles indicate centroids of study groups. Statistical significance of differences between groups based on the resulting model was evaluated using an ANOVA like permutation test.

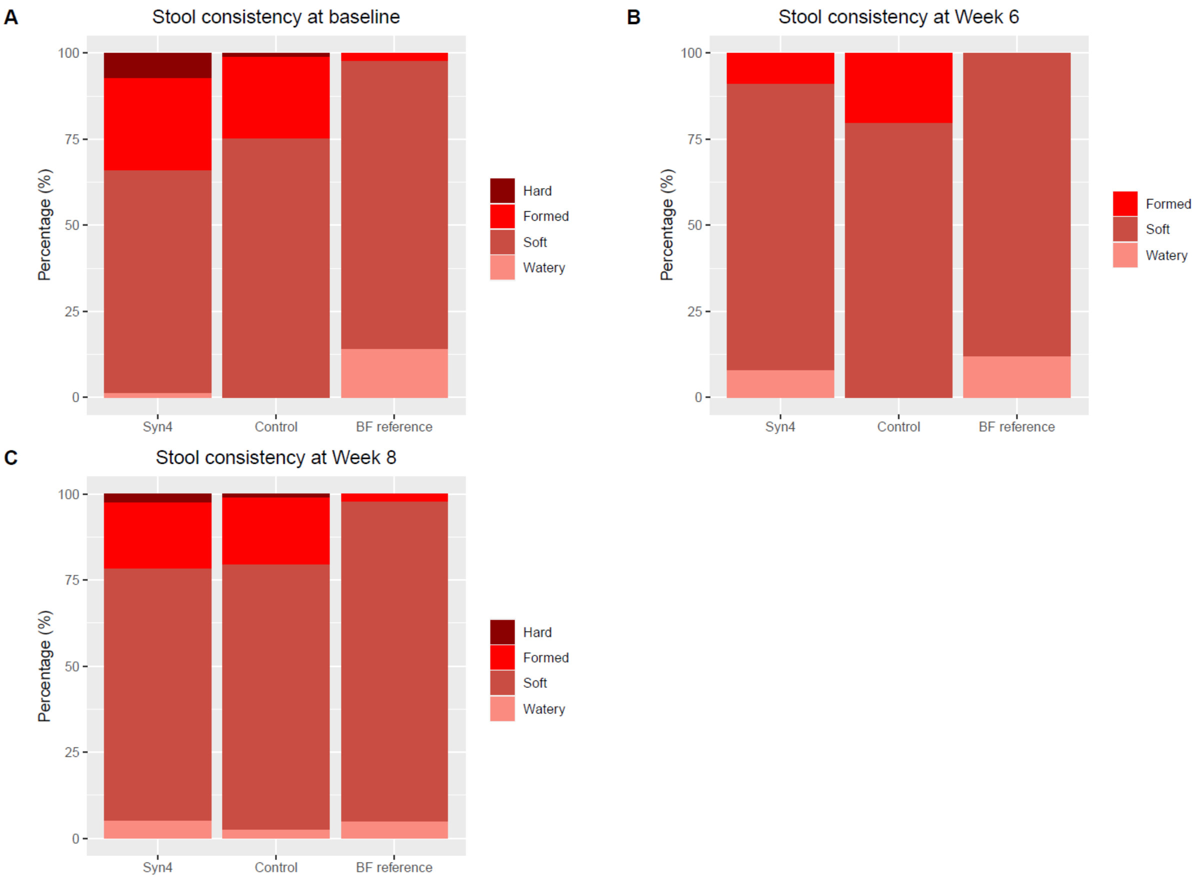

Supplemental Fig.7. Stool consistency types (%) in the Syn4, Control and BF reference groups at baseline (B), Week 6 (C) and Week8 (D) were shown in stacked bar graphs for the control, syn4 and reference groups.

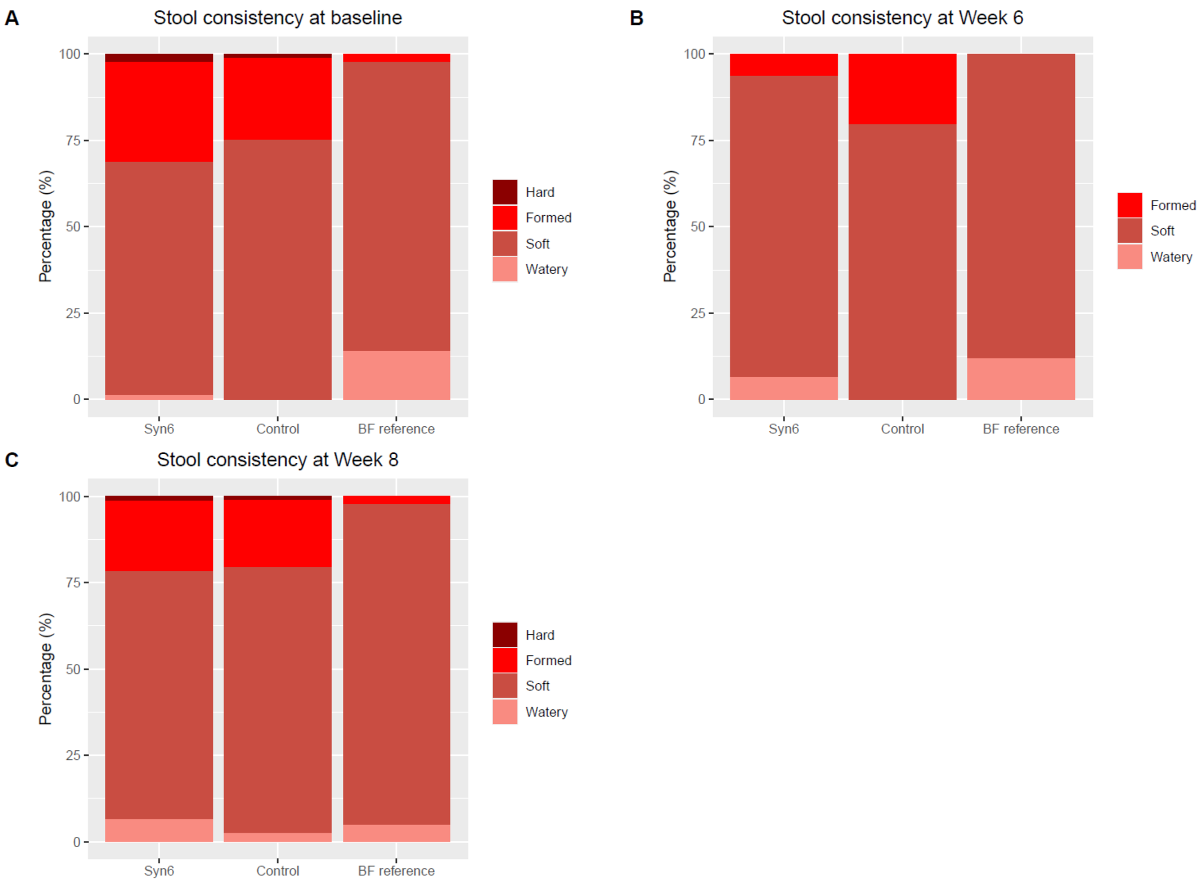

Supplemental Fig.8. Stool consistency types (%) in the Syn6, Control and BF reference groups at baseline (B), Week 6 (C) and Week8 (D) were shown in stacked bar graphs for the control, syn4 and reference groups.

Supplemental Table S1. Summary of linear mixed model for key parameters at Week 6.

LS (Least Squares) estimates of differences in change from baseline between groups, Standard Error Estimates, 95% CI, and P-values for the linear mixed model key parameters measured at week 6 (*Bifidobacterium, Eubacterium rectale-Clostridium coccoides*, pH, L-lactate, Acetate, Propionate, Butyrate).

Key parameter	Difference in change from baseline at 6 weeks	Diff	SE	95% CI	p-value
Bifidobacteria %	(Syn4 Week 6 - Syn4 Baseline) vs. (Control Week 6 - Control Baseline)	15.07	3.46	(8.25, 21.90)	<.0001
	(Syn6 Week 6 - Syn6 Baseline) vs. (Control Week 6 - Control Baseline)	8.54	3.45	(1.74, 15.35)	0.0141
Eubacterium rectale-Clostridium coccoides %	(Syn4 Week 6 - Syn4 Baseline) vs. (Control Week 6 - Control Baseline)	-8,43	1.95	(-12.24, 43.61)	<.0001
	(Syn6 Week 6 - Syn6 Baseline) vs. (Control Week 6 - Control Baseline)	-7,99	1.97	(-11.45, -3.67)	0.0002
pH	(Syn4 Week 6 - Syn4 Baseline) vs. (Control Week 6 - Control Baseline)	-0,75	0.12	(-0.97, -0.52)	<.0001
	(Syn6 Week 6 - Syn6 Baseline) vs. (Control Week 6 - Control Baseline)	0,47	0.12	(-0.70, -0.25)	<.0001
L-lactate	(Syn4 Week 6 - Syn4 Baseline) vs. (Control Week 6 - Control Baseline)	15.63	2.43	(10.84, 20.43)	<.0001
	(Syn6 Week 6 - Syn6 Baseline) vs. (Control Week 6 - Control Baseline)	11.53	2.44	(6.72, 16.33)	<.0001
Acetate %	(Syn4 Week 6 - Syn4 Baseline) vs. (Control Week 6 - Control Baseline)	11.43	1.67	(8.15, 14.71)	<.0001
	(Syn6 Week 6 - Syn6 Baseline) vs. (Control Week 6 - Control Baseline)	8.53	1.68	(5.23, 11.83)	<.0001
Proprionate %	(Syn4 Week 6 - Syn4 Baseline) vs. (Control Week 6 - Control Baseline)	-5,96	1.28	(-8.47, -3.44)	<.0001
	(Syn6 Week 6 - Syn6 Baseline) vs. (Control Week 6 - Control Baseline)	-4,16	1.29	(-6.69, -1.63)	0.0014
Butyrate %	(Syn4 Week 6 - Syn4 Baseline) vs. (Control Week 6 - Control Baseline)	-3,35	0.79	(-4.91, -1.79)	<.0001
	(Syn6 Week 6 - Syn6 Baseline) vs. (Control Week 6 - Control Baseline)	-2,55	0.80	(-4.12, -0.98)	0.0015

Supplemental Table S2. Characteristics of the participants at the beginning of the study.

	Syn4	Syn6	Control	Breast-fed Reference
**Number of subjects**	n = 82	n = 81	n = 84	n = 43
**Gestational age, mean (SD)**	39.00 (1.01)	38.93 (1.13)	38.79 (1.01)	39.03 (0.94)
**Mode of delivery**				
**Vaginal, N (%)**	48 (58.5%)	49 (60.5%)	50 (59.5%)	31 (72.1%)
**C-section, N (%)**	34 (41.5%)	32 (39.5%)	34 (40.5%)	12 (27.9%)
**Gender**				
**Male, N (%)**	42 (51.2%)	47 (58.0%)	48 (57.1%)	20 (46.5%)
**Female, N (%)**	40 (48.8%)	34 (42.0%)	36 (42.9%)	23 (53.5%)
**Feeding pattern**				
**Breast milk, N (%)**	0 (0%)	0 (0%)	0 (0%)	43 (100%)
**Mixed, N (%)**	0 (0%)	0 (0%)	0 (0%)	0 (0%)
**Formula, N (%)**	82 (100%)	81 (100%)	84 (100%)	0 (0%)
**Formula intake ml/day, mean (SD)**	834.50 (234.06)	848.63(200.23)	867.82 (255.54)	-
**Ethnicity**				
**Thai, N (%)**	81 (98.8%)	81 (100.0%)	84 (100.0%)	43 (100.0%)
**Others, N (%)**	1 (1.2%)	0 (0.0%)	0 (0.0%)	0 (0.0%)

Supplemental Table S3. Summary of study discontinuation (Intention-to-Treat).

**Disposition, n (%)**		**Syn 4**	**Syn 6**	**Control**	**Breast Fed Reference**
		n = 82	n = 81	n = 84	n = 43
**Treated**		82 (100.0)	81 (100.0)	84 (100.0)	NA
**Completed the study**		78 (95.1)	78 (96.3)	83 (98.8)	42 (97.7)
**Discontinued the study**		4 (4.9)	3 (3.7)	1 (1.2)	1 (2.3)
	Within 4 weeks	0	1 (1.2)	0	0
	Within 8 weeks	4 (4.9)	1 (1.2)	0	0
	Within 10 weeks	0	1 (1.2)	1 (1.2)	1 (2.3)
**Reason for discontinuation**	Adverse Event	1 (1.2)	1 (1.2)	0	0
	Withdrawal by parent(s)	1 (1.2)	1 (1.2)	1 (1.2)	1 (2.3)
	Lost to follow-up	2 (2.4)	0	0	0
	Protocol violation	0	1 (1.2)	0	0
